# Characterization of primary human T-cell responses to p-phenylenediamine and Bandrowski's base

**DOI:** 10.1186/2045-7022-4-S3-P111

**Published:** 2014-07-18

**Authors:** Andrew Gibson, Kim Seung-Hyun, Lee Faulkner, B Kevin Park, Dean J Naisbitt

**Affiliations:** 1 University of Liverpool, UK; 2 University of Liverpool, MRC Centre for Drug Safety Science, UK

## 

p-Phenylenediamine (PPD) is a component of certain henna tattoo and hair dye formulations. Human exposure to PPD containing products is associated with the development of T-cell mediated allergic contact dermatitis. Antigen-specific T-cells from allergic contact dermatitis patients are activated with either PPD or Bandrowskis base (BB), the metabolized trimer of PPD. In non-allergic individuals T-cells that are activated by BB, but not by PPD, are readily detectable. Antigen-specific T-cells from patients with allergic contact dermatitis show increased secretion of Th2 cytokines. The aim of the current study was to use an in vitro T-cell priming assay that utilizes peripheral blood mononuclear cells from healthy donors to study the activation of memory (CD45RO+) and naïve (CD45RA+) T-cells with PPD and BB. T-cells were cloned from the priming assay and the phenotype and function of antigen-specific cells was compared with T-cells from patients with allergic contact dermatitis. The T-cell priming assay requires cell separation from whole peripheral blood mononuclear cells using magnetic bead sorting, followed by a period of memory/naïve T-cell culture with CD14+ monocyte-derived dendritic cells and PPD or BB. After this, antigen-specific (PPD 2.5-10μM; BB 2.5-10μM) proliferation was measured by [3H]thymidine incorporation and cytokine release by IFNγ, IL-13, and IL-22 ELISpot. Cell phenotype was characterised by flow cytometry. Concentration-dependent BB-specific T-cell proliferative responses were detected with both naïve and memory T-cells from healthy donors. Activation of the T-cells was associated with the secretion of IFNγ, IL-13 and granzyme B. In contrast, PPD-specific T-cells were not detected from either the memory or naïve compartment. T-cell clones generated from the BB-responsive cultures were activated in the presence of BB, but not PPD. As described previously, T-cell proliferative responses and cytokine release to PPD and BB were detected with peripheral blood mononuclear cells from allergic patients and PPD and BB specific clones were generated. In conclusion these data show that BB stimulates pre-existing memory T-cells isolated from allergic patients and healthy donors. Furthermore, BB primes naïve T-cells when the chemical is presented by autologous dendritic cells. In contrast, priming naïve T-cells against PPD failed which suggests an important individual susceptibility factor is missing from the existing T-cell priming assay.

